# A Novel Micromixer That Exploits Electrokinetic Vortices Generated on a Janus Droplet Surface

**DOI:** 10.3390/mi15010091

**Published:** 2023-12-31

**Authors:** Chengfa Wang, Yehui He

**Affiliations:** 1 Department of Marine Engineering, Dalian Maritime University, Dalian 116026, China; 2 Computer Center, The Second Hospital of Dalian Medical University, Dalian 116023, China; yehuihe@163.com

**Keywords:** micromixer, electrokinetic vortices, Janus droplet, zeta potential ratio

## Abstract

Micromixers play a crucial role as essential components in microfluidic analysis systems. This paper introduces a novel micromixer designed by harnessing electrokinetic vortices arising on the surface of a Janus droplet within a microchannel. The Janus droplet is characterized by different polarities of charges on its two sides (upstream part and downstream part). In the presence of a direct current electric field, the droplet’s surface generates electroosmotic flows in opposite directions, resulting in the formation of vortices and facilitating solution mixing. Results from numerical simulations suggest that a better mixing performance of the micromixer is associated with both a higher absolute value of the zeta potential ratio between the downstream and upstream surfaces of the Janus droplet and a larger downstream surface area. Additionally, this study reveals that microchannel dimensions significantly influence the performance of the micromixer. Smaller microchannel widths and heights correspond to a larger mixing index for the micromixer. The micromixer presented in this study features a simple structure, easy fabrication, and holds promising application potential.

## 1. Introduction

Microfluidic technology allows for the integration of fundamental operations, such as sample reactions, separation, and detection, onto microscale chips, enabling rapid sample analysis, and has been widely applied in the fields of biology, medicine, and chemistry [1,2,3,4,5,6]. However, due to the typically low Reynolds numbers in microchannels, fluid convection and diffusion effects are relatively weak, making efficient solution mixing challenging. Therefore, micromixers or fluid-mixing units serve as crucial components in microfluidic devices.

To date, numerous types of micromixers have been reported [7,8,9,10,11]. One type of micromixer utilizes complex channel structures [12,13,14], arrays of obstacles within the channels [15,16], or grooves at the channel walls [17,18,19] to obstruct and disturb fluid flow, thereby enhancing convection and diffusion effects and achieving solution mixing. For example, Enders et al. [13] manufactured five types of micromixers with complex structures using high-definition MultiJet 3D printing and evaluated their mixing performance through numerical simulations and experiments. Chen and Zhao [15] designed a multi-unit obstacle micromixer by placing obstacle arrays at the bottom of a microchannel and studying its mixing efficiency. Results indicated that, compared with a T-type micromixer, this micromixer exhibited higher mixing efficiency. Howell et al. [18] proposed a micromixer with grooves at the top and bottom of a microchannel. The grooves induce the generation of vortices by disturbing the fluid, thereby enhancing the fluid-mixing performance.

Another type of micromixer relies on external energy sources, such as electric fields [20,21,22], magnetic fields [23,24], and acoustic fields [25,26,27], to perturb the solution and facilitate its mixing. As widely recognized, a charged surface in water creates an electric double layer (EDL), and in the presence of a direct current (DC) electric field, electroosmotic flow (EOF) occurs within an EDL. When the charge distribution on the surface is non-uniform, EOF near the surface varies in speed and direction, leading to the formation of electrokinetic vortices that can be employed to perturb the liquid, thereby enhancing the mixing efficiency of the solution. The structures of micromixers based on electrokinetic vortices are typically simple and easily integrable, and as a result, they have been widely researched [28].

Under the influence of an electric field, a metal in water induces zeta potentials with opposite polarities and EOFs with opposite directions, which leads to the formation of electrokinetic vortices near the metal surface. Taking advantage of this phenomenon, Wu and Li [29,30] designed a micromixer and investigated its mixing performance through numerical simulations and experiments. However, under the influence of a strong electric field, the metal surface can exhibit the “Bipolar Electrochemistry” phenomenon [31], which in turn affects the formation of electrokinetic vortices. Non-metallic surfaces with opposite polarity charges can also generate electrokinetic vortices. Erickson and Li [32] explored the formation patterns of electrokinetic vortices in a microchannel with surfaces possessing oppositely charged zeta potentials through numerical simulations and investigated their impact on fluid mixing. Subsequently, Biddiss et al. [33] conducted experimental validations, demonstrating that electrokinetic vortices generated on surfaces with non-uniform zeta potentials significantly enhance fluid-mixing efficiency. Additionally, recent studies have shown that under specific conditions, electrokinetic vortices can also be induced on surfaces with zeta potentials of the same polarity, but different magnitudes [34,35]. Based on this type of electrokinetic vortex, Wang [36] designed a structurally simple micromixer and investigated the key parameters influencing its mixing performance. It is worth noting that non-metallic surfaces typically require surface modification techniques to achieve surfaces with non-uniform charge distribution. For specific shapes and sizes of surfaces, such as microscale cylindrical surfaces, obtaining surfaces with non-uniform zeta potentials through surface modification can be challenging.

Janus droplets are liquid droplets composed of two distinct regions with different properties, and have gained widespread attention in various fields, including drug delivery [37,38] and biomolecule detection [39]. If a droplet has charges of opposite polarity on its two sides, theoretically, the surface of the droplet would also generate electrokinetic vortices. Research indicates that microparticles and nanoparticles tend to adsorb at fluid interfaces [40], and charged particles at the interface undergo movement via a DC electric field [41,42]. Exploiting this phenomenon, Li and Li [43] introduced a certain concentration of nanoparticles into water, allowing them to adsorb onto an oil droplet surface. Subsequently, a DC electric field was applied to induce particle aggregation on one side of the oil droplet. Due to the different charge densities and polarities on the oil droplet and nanoparticle surfaces, a Janus droplet with distinct surface electric properties was obtained. This method allows for the convenient and rapid generation of different types of Janus droplets by varying the type and concentration of nanoparticles.

Currently, there is a scarcity of studies regarding variations in electrokinetic vortices on the surface of Janus droplets and their impact on fluid mixing. Therefore, this paper proposes a micromixer design based on Janus droplets and utilizes numerical simulations to investigate the influence of the electrokinetic vortices formed on a Janus droplet surface on fluid mixing. This research aims to provide a theoretical foundation for subsequent practical applications.

## 2. Micromixer Structure and Simulation for Mixing Performance

**Figure 1:**
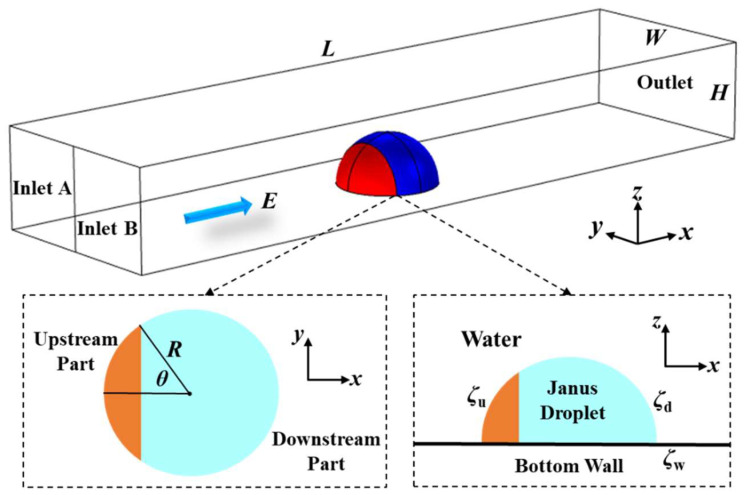
Schematic illustration of the micromixer structure.

Figure 1 illustrates the micromixer structure designed in this study. At the bottom of the rectangular microchannel, there is an adhered Janus droplet with a radius of *R* = 10 μm. The dimensions of the microchannel are defined as length (*L*), width (*W*), and height (*H*). In this study, the droplet radius *R* was set as the characteristic length. A microchannel has two inlets: Inlet A and Inlet B. A water solution fills the microchannel, and a DC electric field is imposed on the water. The longer the microchannel, the longer the time the fluid flows in the channel, resulting in an extended mixing time. Therefore, it is easy to understand that, under the same conditions, a longer microchannel leads to better fluid mixing at the channel outlet. Hence, in this study, we chose a microchannel length of *L* = 20*R* as a typical example for investigation.

As shown in Figure 1, the Janus droplet adhering to the channel’s bottom wall is divided into two parts: the upstream part and the downstream part. In this study, the upstream part was set to carry negative charges, while the downstream part was set to carry positive charges. The zeta potentials on the surfaces of these two parts are defined as *ζ*_u_ and *ζ*_d_, respectively. The parameter *α* = *ζ*_d_/*ζ*_u_ represents the zeta potential ratio between the downstream surface and the upstream surface. The zeta potential on the channel wall is denoted as *ζ*_w_. In this study, constant and identical zeta potentials were assigned to the microchannel wall and the Janus droplet’s upstream surface; i.e., *ζ*_w_ = *ζ*_u_ = −20 mV.

The parameter *θ* is defined to illustrate the size relationship between the upstream part and downstream part. A smaller *θ* indicates a larger area occupied by the downstream surface of the Janus droplet. Additionally, the three-phase contact angle formed by the Janus droplet adhering to the bottom wall in water is related to the properties of the droplet and the solid wall [44,45]. To simplify the model, this study used a three-phase contact angle of 90° as a typical case for investigation.

Due to the opposite polarity of the charges on the two surfaces of the Janus droplet, after a DC electric field is imposed within the channel, the Janus droplet’s surface generates two EOFs in opposite directions, leading to the formation of vortices near the droplet. The vortices disturb the fluid, thereby promoting the mixing of the solution within the microchannel. Therefore, this simulation model includes three physical fields: electric field, flow field, and concentration field. The specific governing equations and boundary conditions are as follows.

### 2.1. Electric Field

The electric potential (*V*) distribution of the DC electric field in the microchannel follows Laplace’s equation:

(1) $$\nabla^{2}V=0$$

The relationship between the electric potential and the local electric field strength (***E***) is as follows:

(2) E=−∇V

The microchannel walls and the Janus droplet’s surface adhere to the insulating boundary conditions; that is,

(3) n⋅E=0

where ***n*** is the unit normal vector on the boundaries.

The channel outlet is grounded, and the same constant voltage *V*_in_ is applied to Inlet A and Inlet B, maintaining an electric field strength in the channel at *V*_in_/*L* = 1000 V/m.

### 2.2. Flow Field

In the steady state, the flow field distribution in the channel is governed by the following equations:

(4) $$\rho U\cdot \nabla U=-\nabla p+\mu \nabla^{2}U$$

(5) ∇⋅U=0

where *ρ* = 1000 kg/m^3^ and *μ* = 0.001 Pa·s represent the density and viscosity of the aqueous solution, respectively, U is the flow velocity of the solution, and *p* denotes pressure.

In the micromixer designed in this study, the fluid flow relies entirely on the EOF generated at the walls. Therefore, the inlet and outlet pressures of the channel were both set to 0. As the thickness of an EDL is much smaller compared to the dimensions of the channel and the Janus droplet, the flow field distribution in the EDL can be neglected. Thus, to simplify the model and reduce computational complexity, the theoretical calculation formula for EOF velocity (Helmholtz–Smoluchowski Equation [46]) was applied as the boundary condition at the corresponding boundaries as follows.

On the channel walls:

(6) $$U=-\frac{\varepsilon_{0}\varepsilon \zeta_{w}}{\mu }E$$

On the upstream surface of the Janus droplet:

(7) $$U=-\frac{\varepsilon_{0}\varepsilon \zeta_{u}}{\mu }E$$

On the downstream surface of the Janus droplet:

(8) $$U=-\frac{\varepsilon_{0}\varepsilon \zeta_{d}}{\mu }E$$

where *ε*_0_ = 8.85 × 10^−12^ F/m is the vacuum permittivity, and *ε* = 80 is the solution relative permittivity.

### 2.3. Concentration Field

This study assumed that the solution concentration remains unaffected by any reactions. Therefore, in the steady state, the concentration distribution of the aqueous solution in the microchannel follows the following governing equation:

(9) ∇⋅(−D∇c)+U⋅∇c=0

where *D* = 10^−11^ m^2^/s is the diffusion coefficient of the solute in water, and *c* represents the solution concentration.

As the solute cannot pass through the boundaries, a no-flux condition is imposed on the channel walls and the droplet’s surface, which is expressed as:

(10) −n⋅(−D∇c+Uc)=0

At the boundaries of Inlet A and Inlet B, the solution concentration was set to one mol/m³ and zero, respectively. The solute is carried out of the outlet by flowing liquid, indicating the dominance of convection in solute transport. Therefore, the outlet boundary condition is:

(11) n⋅D∇c=0

In the aforementioned three-dimensional simulation model, the electric field, flow field, and concentration field were mutually coupled, and their solution was carried out using the finite element analysis software COMSOL Multiphysics 5.4 (COMSOL Co., Ltd., Shanghai, China).

## 3. Results and Discussion

### 3.1. Flow Field and Concentration Field in the Microchannel

**Figure 2:**
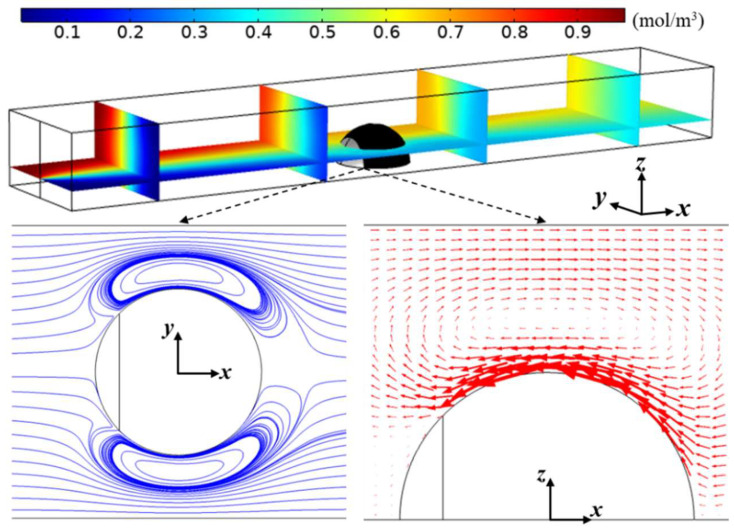
Distribution of the concentration field and flow field in the microchannel. Solid lines represent streamlines of the flow field, while arrows illustrate the flow’s velocity and direction. The contour of the electrokinetic vortices is illustrated through the streamline distribution in the microchannel. The rotation direction and speed of the electrokinetic vortices are represented by the size and direction of the arrows.

Figure 2 illustrates the distribution of the concentration field and flow field in the microchannel under steady-state conditions. In the figure, the channel width is W* = *W*/*R* = 3.5, the channel height is H* = *H*/*R* = 2, and the zeta potential ratio *α* between the downstream and upstream surfaces of the Janus droplet is set to −5, with an angle *θ* = 45°. As observed in the figure, when subjected to a DC electric field, the negatively charged upstream surface of the Janus droplet generated an EOF directed towards the negative pole of the electric field, while the positively charged downstream surface of the Janus droplet generated an EOF directed towards the positive pole of the electric field. The interaction between the two oppositely-directed EOFs led to the formation of clockwise-rotating vortices near the downstream surface of the Janus droplet, as shown in Figure 2. As the solution flowed past the Janus droplet, the vortices disturbed the liquid, thereby promoting the mixing of the solution.

This study defined the ratio *γ* of the minimum concentration at the channel outlet to the ideal mixed concentration as the mixing index to quantitatively evaluate the micromixer’s mixing performance. The minimum concentration at the channel outlet can be directly obtained through the software. Subsequent sections focus on investigating the influence of different factors on this parameter.

### 3.2. Effect of the Zeta Potential Ratio of the Janus Droplet’s Surface on Mixing Efficiency

**Figure 3:**
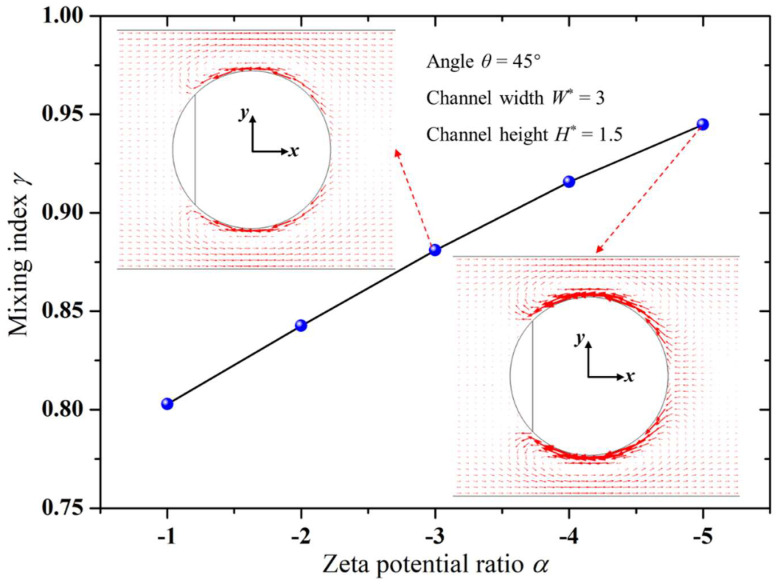
Variation in mixing index with zeta potential ratio of Janus droplet surface.

Figure 3 depicts the variation in the mixing index (*γ*) with the zeta potential ratio (*α*) between the downstream and upstream surfaces of the Janus droplet. It was evident that the larger the absolute value of the zeta potential ratio *α*, the higher the mixing index *γ*, which indicated better mixing performance. This was attributed to the increasing absolute value of the zeta potential ratio *α*, which resulted in a larger zeta potential on the downstream surface of the Janus droplet. Consequently, the EOF velocity on the downstream surface became faster, as shown in Figure 3, leading to a higher rotation speed of the electrokinetic vortices generated near the downstream surface. This enhanced rotation contributed to more effective agitation of the solution, thus improving the mixing performance. Therefore, the mixing index increased with the increasing absolute value of the zeta potential ratio *α*.

In experimental studies, Janus droplets can be prepared by choosing nanoparticles with different charges, thereby altering the zeta potential on the downstream surface of the Janus droplet and consequently adjusting the electrokinetic vortices generated on the downstream surface.

### 3.3. Impact of Zeta Potential Distribution along Janus Droplet Surface

**Figure 4:**
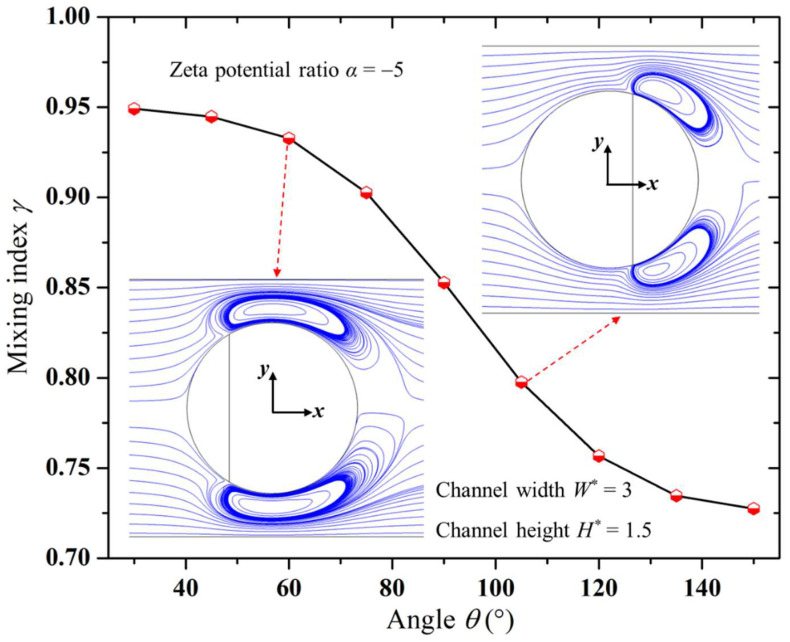
Variation in mixing index with angle of Janus droplet.

Figure 4 shows the impact of the angle (*θ*) on the mixing performance of the micromixer. It is evident from the figure that, under the same conditions, a smaller angle *θ* corresponded to a larger mixing index, indicating better mixing performance of the micromixer. The reason for this is as discussed earlier; the downstream surface of the Janus droplet was positively charged, resulting in an EOF opposite to the direction of the EOFs generated by the droplet upstream and the channel wall. This led to the formation of electrokinetic vortices near the downstream surface of the Janus droplet, as depicted in Figure 2. A smaller angle *θ* implies a smaller upstream part and a larger downstream part of the Janus droplet, as shown in Figure 1. As the angle *θ* decreased, the vortices formed near the downstream surface of the Janus droplet became larger, as depicted in Figure 4. This, in turn, produced stronger disturbances in the solution flowing through the droplet, leading to a better mixing performance of the micromixer.

In experimental studies, increasing the concentration of nanoparticles can lead to more nanoparticles adsorbing onto the droplet surface [43]. This can be used to prepare Janus droplets with a larger downstream area, thereby adjusting the electrokinetic vortices generated on the downstream surface.

### 3.4. Influence of Microchannel Dimensions on Mixing Performance

**Figure 5:**
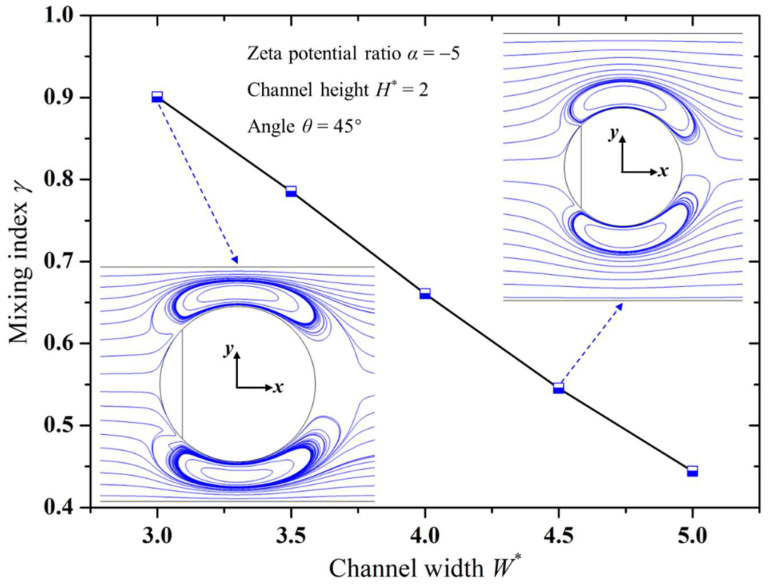
Variation in mixing index with microchannel width.

**Figure 6:**
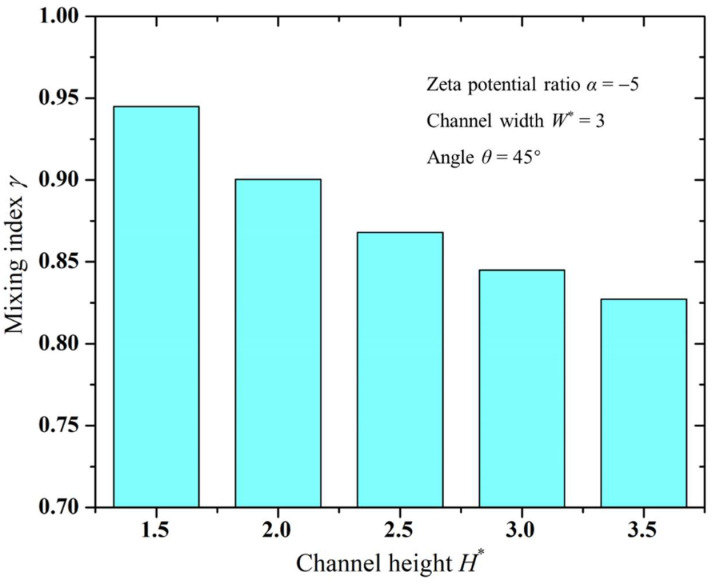
Variation in mixing index with microchannel height.

Figure 5 and Figure 6 illustrate variation trends in the mixing index with respect to the channel’s width and height, respectively. It is clear from these figures that, under the same conditions, smaller channel widths and heights led to a better mixing performance in the micromixer. With the reduction in channel width, the squeezing effect of the channel sidewalls on the vortices formed on the downstream surface of the Janus droplet became stronger, resulting in a faster rotation speed of the vortices. On the other hand, the decrease in channel width narrowed the gap between the droplet and the channel sidewalls, allowing more solution to flow through the vortices. Therefore, a smaller channel width enhances the stirring effect of the electrokinetic vortices, resulting in improved mixing performance, as shown in Figure 5. Similar to the effect of the channel width, as the channel height decreased, the electrokinetic vortices formed at the top of the Janus droplet became stronger in disturbing the solution, thereby enhancing the mixing index, as illustrated in Figure 6. Additionally, comparing Figure 5 and Figure 6 reveals that, relative to the influence of channel width, the impact of channel height on mixing efficiency was relatively smaller. This is mainly because changes in channel width simultaneously alter the effects of both channel sidewalls on the electrokinetic vortices, whereas changes in channel height affect only the impact of the top channel wall on the electrokinetic vortices.

The above findings provide a theoretical basis for the subsequent design of micro-mixer dimensions. Compared to the size of Janus droplets, the height and width of the microchannel cannot be designed too large; otherwise, the mixing efficiency of the micromixer will be significantly compromised.

The design of micromixers based on the disturbance of fluid flow through complex channel structures to achieve efficient fluid mixing is an important approach. However, micromixers with complex structures pose challenges in terms of fabrication and integration. In this study, a proposed micromixer was designed based on the electrokinetic vortices formed on the surface of a Janus droplet. This design is structurally simple, easy to fabricate, and conducive to integration into microfluidic chips.

It is important to note that, for simplification, we chose the case of a Janus droplet in water with a 90° three-phase contact angle on the bottom surface as a typical example in our research. Future studies can delve into the impact of droplet three-phase contact angles on the surrounding electrokinetic vortices and fluid mixing. Furthermore, this study assumed that the Janus droplet’s shape remained unchanged. However, in practical applications, high DC electric field intensities may cause deformation of droplets, affecting the formation of electrokinetic vortices and, consequently, leading to ineffective fluid mixing. Therefore, further investigation is needed to explore the critical DC electric field intensity that causes droplet deformation, providing theoretical insights for subsequent practical applications.

## 4. Conclusions

This study utilized electrokinetic vortices generated by a charged asymmetric Janus droplet in the presence of a DC electric field to design a structurally simple micromixer. Through numerical simulations, variations in the electrokinetic vortex and its impact on the micromixer mixing efficiency were studied, leading to the following main conclusions:

(1) The downstream surface of a Janus droplet with a larger positive zeta potential, compared to the upstream surface with a negative zeta potential, results in a stronger electrokinetic vortex, thereby improving mixing efficiency. This provides a reference for the selection of nanoparticle types required for the preparation of Janus droplets.

(2) An increase in the size of the downstream part of a Janus droplet leads to a larger electrokinetic vortex on its surface, enhancing the mixing efficiency of the solution. This can guide the selection of nanoparticle concentrations for the preparation of Janus droplets.

(3) Smaller cross-sectional dimensions of the microchannel lead to a stronger disturbance of the solution by the electrokinetic vortices, consequently improving the mixing performance. This provides a reference for the design of the structural dimensions of the micromixer.

This research provides theoretical insights into the practical applications of electrokinetic vortices on Janus droplet surfaces in fluid mixing.

## Data Availability

Data are contained within the article.
